# Non-pharmacological interventions for improving sleep in people living with HIV: a systematic narrative review

**DOI:** 10.3389/fneur.2023.1017896

**Published:** 2023-11-20

**Authors:** Jingjing Meng, Chunyuan Zheng, Honghong Wang, Maritta Välimäki, Min Wang

**Affiliations:** ^1^School of Nursing, Anhui Medical University, Hefei, Anhui, China; ^2^Xiangya Nursing School, Central South University, Changsha, Hunan, China; ^3^Xiangya Center for Evidence-Based Practice & Healthcare Innovation: A Joanna Briggs Institute Affiliated Group, Xiangya Nursing School, Central South University, Changsha, Hunan, China; ^4^Department of Nursing Science, University of Turku, Turku, Finland; ^5^The Institute of HIV/AIDS, The First Hospital of Changsha, Hunan, China

**Keywords:** sleep, non-pharmacological interventions, sleep measurements, people living with HIV, narrative review

## Abstract

**Background:**

Sleep disturbances are common in people living with Human Immunodeficiency Virus (HIV) and may lead to poor adherence to antiretroviral therapy and worsen HIV symptom severity. Due to the side effects of pharmacotherapy for sleep disturbances, there is more room for non-pharmacological interventions, but knowledge of how these non-pharmacological interventions have been used to improve sleep in people living with HIV (PLWH) is still missing.

**Objective:**

To investigate the content of non-pharmacological interventions, sleep measurements, and the impact of these interventions on improving sleep in PLWH.

**Methods:**

Following PRISMA guidelines, we conducted a systematic search on PubMed, EMBASE, Cochrane Central Registry of Controlled Trials, Cumulative Index to Nursing and Allied Health Literature, Web of Science, China National Knowledge Infrastructure, Wanfang Data, and China Biology Medicine disc. Non-pharmacological interventions for improving sleep in PLWH were included, and study quality was assessed using the Joanna Briggs Institute (JBI) critical appraisal checklists. We performed a narrative approach to synthesize the data to better understand the details and complexity of the interventions.

**Results:**

Fifteen experimental studies in three categories for improving sleep in PLWH were included finally, including psychological interventions (components of cognitive-behavioral therapy for insomnia or mindfulness-based cognitive therapy, *n* = 6), physical interventions (auricular plaster therapy, acupuncture, and exercise, *n* = 8), and elemental interventions (speed of processing training with transcranial direct current stimulation, *n* = 1). Wrist actigraphy, sleep diary, and self-reported scales were used to measure sleep. Psychological interventions and physical interventions were found to have short-term effects on HIV-related sleep disturbances.

**Conclusions:**

Psychological and physical interventions of non-pharmacological interventions can potentially improve sleep in PLWH, and the combination of patient-reported outcomes and actigraphy devices can help measure sleep comprehensively. Future non-pharmacological interventions need to follow protocols with evidence-based dosing, contents, and measures to ensure their sustainable and significant effects.

## 1 Introduction

Since the introduction of antiretroviral therapy (ART), mortality among persons with Human Immunodeficiency Virus (HIV) has declined substantially, and HIV infection has transformed into a controllable chronic disease (1). Still, people living with HIV (PLWH) suffer from many chronic conditions, and one is poor sleep quality (2–4). The overall prevalence of self-reported sleep disturbances in PLWH is 58.0% (5). The pathophysiology of sleep disturbances in PLWH is still not well-understood but may be related to the long duration of HIV infection (6), ART regimen containing nonnucleoside reverse transcriptase inhibitors such as efavirenz (2), and opportunistic infections of the central nervous system caused by HIV (7). Due to the sexual transmission route of HIV, PLWH suffers from varying degrees of psychological stress, and sleep disturbances are associated with multiple psychological factors such as depression (6) and HIV-related stigma (8). Even virologically controlled HIV infection is still associated with increased inflammation and immune cell activation and may affect circadian rhythms and sleep architecture through the action of cytokines (9–11). Regardless of the pathophysiological mechanism, sleep disturbances can contribute to a series of adverse consequences for PLWH. Individuals with poorer sleep quality have lower adherence to HIV medication and higher self-reported HIV symptom severity (12). Evidence suggests that higher levels of sleep disturbances in PLWH are closely related to lower CD4^+^T cell count and higher HIV viral load (13, 14). In addition, sleep disturbances may further contribute to fatigue (15), depression (16), impaired cognitive function (17), increased risk of cardiovascular disease (18), and reduced quality of life in PLWH (19). Given the high prevalence and negative impact of sleep disturbances in PLWH, it is necessary to develop appropriate intervention strategies to address the various biological, environmental, and psychological factors that may affect sleep in PLWH.

The current clinical practice guidelines recommend using pharmacological and non-pharmacological therapies to manage sleep disturbances (20). However, side effects of pharmacological therapies, including dependence and interaction with ART, have also been reported (21, 22). PLWH requires lifelong ART, while the co-use of sleep medications might increase the burden of liver detoxification, and the potential interaction between sleep medications and ART regimen needs to be considered when using pharmacological therapy (22). In addition, the efficacy of sleep medications decreases with prolonged use and even disappears when the sleep medication is discontinued (21). Non-pharmacological interventions have also been recommended. Non-pharmacological interventions are science-based and non-invasive interventions for human health, which are intended to prevent, care for, or treat health problems and are associated with biological and/or psychological processes identified in clinical studies (23). Given the effects of non-pharmacological interventions on sleep-related outcomes, the European Sleep Research Society, for example, has recommended cognitive behavioral therapy for insomnia (CBT-I), light therapy, and exercises for insomnia therapy (24).

We systematically searched and found four existing systematic reviews on sleep problems in PLWH. Wu et al. (5) and Chaponda et al. (25) have systematically examined the pooled prevalence of sleep disturbance in HIV infection, found sleep disturbance was a significant burden for PLWH, and emphasized the significance of regular assessment of sleep in PLWH. Reid and Dwyer (26) reviewed 29 studies with a wide variation in both study design and quality and found limited evidence for the effect of specific treatments for insomnia in HIV infection. Voss et al. (27) analyzed the efficacy of nurse-led symptom management interventions for HIV-related fatigue and sleep disturbances, which identified seven nurse-led interventions targeting either biological pathways or sleep hygiene behaviors. However, this study could not recommend with certainty any symptom management strategies to release sleep disturbances in PLWH. There is still a lack of systematic reviews and detailed descriptions of non-pharmacological interventions for HIV-related sleep disturbances, which affects the intervention implementation and dissemination. Having a better understanding of the content of these non-pharmacological interventions to support sleep among PLWH might help us improve the reproducibility of interventions and provide clinical practice recommendations for the future in improving sleep in PLWH (28).

This systematic review aimed to investigate the content, measurements, and impact of non-pharmacological interventions for improving sleep in PLWH. The following review questions were asked: (1) What is the content of the non-pharmacological interventions used to improve sleep of PLWH? (2) What measures have been used to assess the impact of non-pharmacological interventions on sleep in PLWH? and (3) What is the possible impact of non-pharmacological interventions to improve sleep among PLWH? The knowledge will be used to decide whether new studies related to the impact of non-pharmacological studies are still needed.

## 2 Methods

The Preferred Reporting Items for Systematic Reviews and Meta-Analyses (PRISMA) 2020 statement (29) and Synthesis Without Meta-analysis (SWiM) guideline (30) were followed in designing and reporting the narrative review.

### 2.1 Eligibility criteria

Study eligibility criteria were set based on the structured PICOS principles (31). The inclusion and exclusion criteria were as follows. (1) Population: PLWH aged 18 and above without other severe physical complications. We excluded PLWH with central respiratory disease, such as sleep apnea syndrome, as this population requires specialized medical treatment (32). (2) Intervention: non-pharmacological interventions for improving sleep or alleviating sleep disturbances, insomnia, or sleep-related symptoms, such as psychotherapy, health education, and exercise (33). We excluded studies that did not aim to improve sleep but only used sleep indicators as secondary outcomes. (3) Comparison: any comparison interventions or no control group. (4) Outcomes: sleep-related outcomes (such as sleep quality, sleep efficiency, and sleep disturbances) reported by patients or measured by assistive devices. (5) Study design: experimental or quasi-experimental studies that measured pre-and post-intervention data. Only the articles with the complete information would be included for duplicate articles.

### 2.2 Information sources

A systematic literature search was conducted on eight electronic databases, including PubMed, EMBASE, Cochrane Central Registry of Controlled Trials (CENTRAL), Cumulative Index to Nursing and Allied Health Literature (CINAHL), Web of Science, China National Knowledge Infrastructure (CNKI), Wanfang Data, China Biology Medicine disc (CBM). To find missing information on the included trials, we searched for interventional studies on clinicaltrials.gov. We also checked whether there were possible ongoing reviews in PROSPERO. In addition, we manually conducted a hand search using Google Scholar to check the citing and cited reference lists of initially included studies from databases for potential inclusion. Finally, the PubMed “Similar articles” search (34) was conducted to identify relevant articles of included articles.

The retrieval of all databases started from their inception date, and no restriction on language was applied to the search process. The first search was performed on June 29th, 2021, and updated on June 14th, 2022.

### 2.3 Search strategy

Focusing on the critical concepts of sleep, HIV/AIDS, non-pharmacological interventions and their synonyms, the PubMed search terms and thorough keywords strings mainly included the following subject heading: (HIV OR human immunodeficiency virus OR AIDS OR acquired immunodeficiency syndrome) AND (Sleep OR Sleep quality OR Insomnia OR Sleep disorder OR Sleep disturbance OR Sleep Initiation and Maintenance Disorders) AND (Non-pharmacological intervention OR Non-pharmacological therapies OR Behavior intervention OR Behavior Therapy OR Psychotherapy OR Complementary Therapies OR Music Therapy OR Cognitive Behavioral Therapy OR Sleep Hygiene OR Stimulus Control OR Sleep restriction OR Relaxation). The search terms were adapted for the other seven electronic databases. Detailed search strategies for all databases were displayed in Supplementary material 1.

### 2.4 Selection process

The study selection process was based on the PRISMA flow diagram (35). All retrieved records were managed using Endnote (version X8). First, duplicate studies were removed electronically using Endnote's “find duplicate” strategy and then manually. Three reviewers (JM, CZ, HW) independently screened the titles and abstracts of the first 50 records and discussed the inconsistencies until they reached a consensus. Next, two reviewers (JM and CZ) independently screened titles and abstracts. Any discrepancy regarding whether the abstract should be included was solved through discussion with a third reviewer (HW). After discarding the irrelevant studies, the full texts were reviewed by JM and CZ independently. Again, any discrepancy was discussed with a third reviewer (HW) for consensus.

### 2.5 Data items and data collection process

A standardized extraction form was designed to describe the characteristics of the included studies, including the following information: (1) publication details: name of the first author, year, country, and design; (2) participant characteristics: mean age (standard deviation), and sample size. In addition, whether to receive antiretroviral therapy should also be described because the therapy might affect the patient's sleep quality (4).

To answer the review questions, the data from each included article were extracted in a specific table or were described separately. First, the content of the non-pharmacological interventions for improving sleep in PLWH was extracted using the template for intervention description and replication (TIDieR) checklist and guide (28) with 12 items [brief name (the name or a phrase that describes the intervention), why, what (materials and procedure), who provided, how, where, when and how much, tailoring, modifications, how well (planned and actual)]. Second, measurement tools used to assess the impact of non-pharmacological interventions on sleep in PLWH were extracted. Third, the impact of non-pharmacological interventions to improve sleep was summarized. The data were extracted by one reviewer (JM) independently and checked by another reviewer (CZ). Any discrepancy was discussed with the third reviewer (HW).

### 2.6 Study risk of bias assessment

The Joanna Briggs Institute (JBI) critical appraisal checklists for randomized controlled trials (RCTs) and quasi-experimental studies were applied to assess the methodological quality of included studies (36). The assessment was conducted independently by two authors (JM and HW). Any disagreement was resolved by discussion and consultation with a third reviewer (CZ).

### 2.7 Synthesis methods

We constructed structured tables to examine variation in results across studies based on intervention components, study design, and relevant factors. Moreover, we used a narrative approach to synthesize the data to better understand the details and complexity of the interventions.

## 3 Results

### 3.1 Study selection

**Figure 1:**
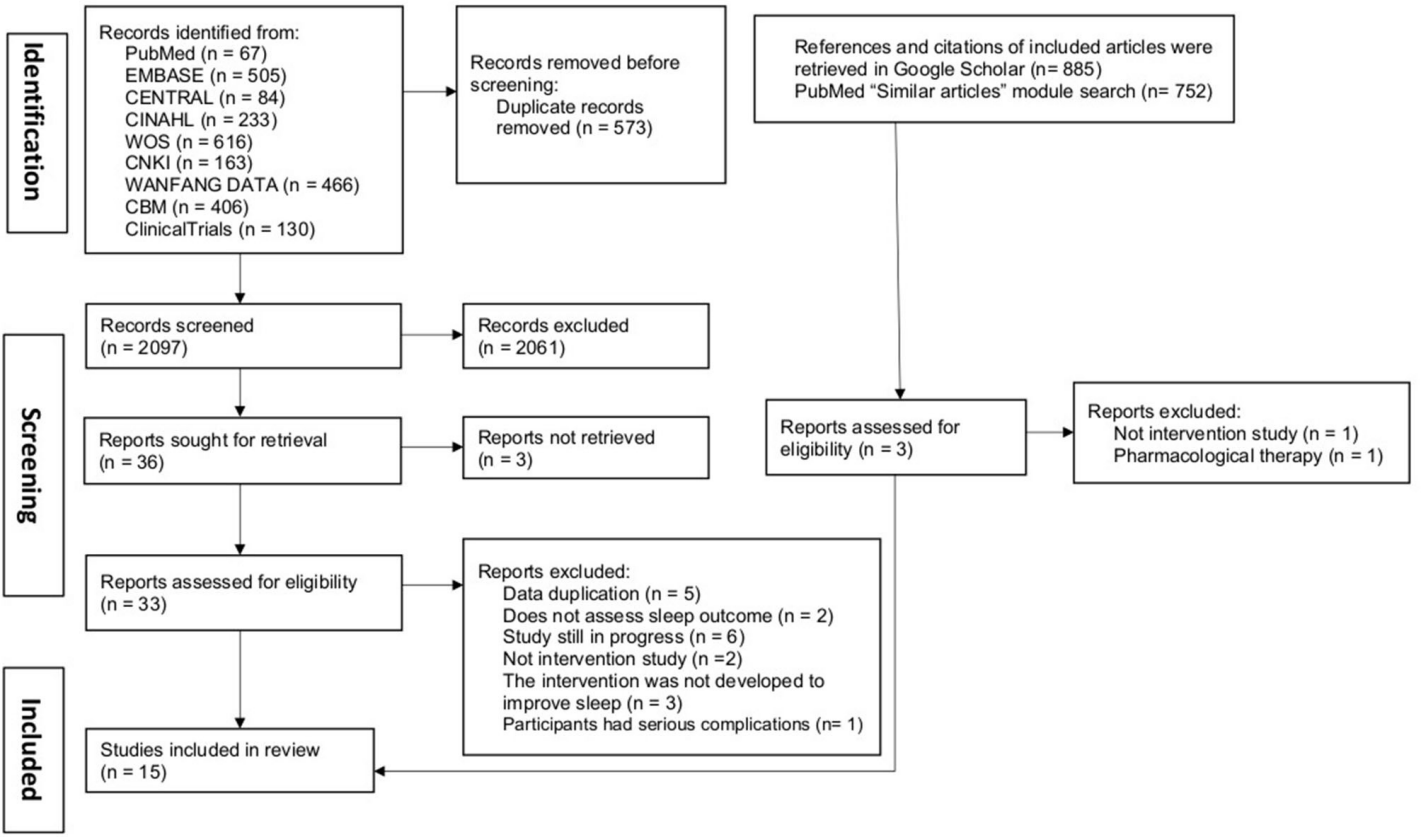
Flow diagram of the study selection process.

A total of 2,670 records were identified by retrieving electronic databases; additional 885 records were identified from the cited and citing references of initially included articles from databases, and 752 records in PubMed were identified from similar articles of final included studies. After 573 duplicated records were removed, 2,097 records remained, of which 2,061 were excluded after titles and abstracts screening. The remaining 36 articles with full text were reviewed according to eligibility criteria. Further, 19 articles were excluded due to data duplication, did not assess sleep outcomes, interventions were not developed for improving sleep, and other reasons. Finally, 15 articles were included in this systematic narrative review. The detailed study selection process based on the PRISMA flow diagram is presented in Figure 1.

### 3.2 Study characteristics

A summary of the included studies is presented in Table 1. All included studies were published between 2001 to 2021. The studies were conducted in America (*n* = 7), China (*n* = 6), and Iran (*n* = 2). Nine articles were published in English (37–44, 51), and the remaining six studies were published in Chinese (45–50). Study designs included RCTs (*n* = 9) and pretest-posttest quasi-studies (*n* = 6). Two RCTs were three-arm studies (41, 49), and the remaining seven RCTs were two-arm studies (38, 40, 42, 46–48, 51).

**Table 1:** Study characteristics.

**References**	**Country**	**Study design**	**Mean age (SD)**	**Sample size**	**Received ART or not**	**Interventions**	**Sleep measurement(s)**	**Impact of intervention**	**Quality score**
Buchanan et al. (37)	America	one-arm quasi	46.0 (–)	22	Yes	(1) Sleep restriction; (2) Stimulus control; (3) Circadian mechanisms discussion; (4) Sleep hygiene	ISI; PSQI; Sleep diary; PROMIS Sleep-related impairment; SHPS; SDQ	Insomnia symptom severity↓ Sleep quality↑ Sleep-related impairment↓ Sleep disturbances↓ Total sleep time↑ Sleep onset latency↓ Wake after sleep onset↓ Sleep efficiency↑ No significant change in fatigue, time in bed, and variability of bedtimes	7
Webel et al. (38)	America	two-arm RCT	Intervention: 49.1 (7.4); Comparison: 47.8 (6.4)	Intervetion:21; Comparison:22	95% of participants received	Sleep hygiene; Behavioral modification strategies	Wrist actigraphy; PROMIS Sleep Disturbance and Sleep-Related Impairments	No significant changes in sleep outcomes	9
Hudson et al. (39)	America	One-arm quasi	40.4 (6.3)	32	Yes	Educational and behavioral set of sleep-promoting behaviors based on principles of sleep hygiene	PSQI; Wrist actigraphy;GSDS	General sleep disturbance↓ Sleep maintenance↑ Circadian rhythm parameters↑ The perception of sleep↑ No significant change in total sleep time at night, sleep onset, number of awakenings, or duration of awakenings	7
Dreher (40)	America	Two-arm RCT	Total: 41.28 (7.87)	Intervention: 54; Comparison: 66	Yes	Avoid all caffeine sources for 30 days	PSQI	A 35% significant improvement in sleep among intervention group subjects	5
Alikhani et al. (41)^*^	Iran	Three-arm RCT	TRAZ: 37.71 (6.38) SHT: 39.78 (6.26) SHT + TRAZ: 41.27 (6.44)	TRAZ: 25 Sleep hygiene: 25 Sleep hygiene + TRAZ: 25	Unclear	Principles of sleep hygiene, such as having a regular sleep schedule, getting rid of “bad” thoughts to fall asleep	PSQI; ISI; ESS	Sleep quality↑ Sleep disturbances↓ Daytime functioning↑ No significant changes in daytime sleepiness and symptoms of insomnia	9
Molavi et al. (42)	Iran	Two-arm RCT	Intervention: 35.7 (7.42) Comparison: 37.8 (9.1)	Intervention: 15 Comparison: 15	Yes	Mindfulness-based cognitive therapy	PSQI	Sleep quality↑	7
Hixon et al. (43)	America	Two-arm quasi	PLWH: 57.3 (5.7) Uninfected controls: 60.2 (6.8)	PLWH: 32 Uninfected controls: 37	Yes	Supervised exercise intervention	PSQI; Wrist-worn actigraphy	No significant changes in sleep outcomes	7
Phillips and Skelton (44)	America	One-arm quasi	41.7 (6.6)	23	Unclear	Individualized acupuncture treatments that focused on specific needs and symptoms that the individual was experiencing	PSQI; Wrist actigraphy; CSQI	Sleep quality↑ The amount of sleep in minutes↑ Sleep percentage↑ Sleep ratio↑ The number of minutes spent awake↓ Wake percentage↓ Total sleep time↑ Subjective sleep quality measured by CSQI↑ No significant change in mid-sleep awakenings, the amount of time spent awake after each mid-sleep awakening, and sleep latency	7
Chen et al. (45)	China	One-arm quasi	Not reported	272	Yes	Auricular plaster therapy	PSQI and its subscales	Sleep quality↑ Sleep duration↑ Sleep efficiency↑ Sleep latency↓ Sleep disturbances↓ Use of medications↓ Daytime dysfunction↓	4
Chen (46)	China	Two-arm RCT	Intervention: 44.05 (2.091) Comparison: 40.35 (1.865)	Intervention: 40 Comparison: 40	Yes	Auricular plaster therapy	PSQI	Sleep quality of intervention group↑	10
Zhang (47)	China	Two-arm RCT	Intervention: 60.85 (10.83) Comparison: 61.03 (11. 01)	Intervention: 43 Comparison: 43	Yes	Auricular plaster therapy with emotional Intervention	PSQI	Sleep quality of intervention group↑	8
Sun (48)	China	Two-arm RCT	Intervention: 59.01 (9.58) Comparison: 58.65 (9.65)	Intervention: 29 Comparison: 29	Unclear	Auricular plaster therapy with emotional intervention	PSQI	Sleep quality of intervention group↑	8
Zhang (49)	China	Three-arm RCT	Intervention 1: 47.98 (10.60) Intervention 2: 51.56 (9.93) Comparison: 50.46 (11.57)	Intervention 1: 40 Intervention 2: 41 Comparison: 40	Unclear	Intervention 1: auricular plaster therapy with emotional intervention; Intervention 2: auricular plaster therapy	PSQI and its subscales	Sleep quality↑ Sleep duration↑ Sleep efficiency↑ Sleep latency↓ Sleep disturbances↓ Use of medications↓ Daytime dysfunction↓	7
Li et al. (50)	China	One-arm quasi	Not reported	68	Unclear	Auricular plaster therapy	PSQI and its subscales	Sleep quality↑ Sleep duration↑ Sleep efficiency↑ Sleep latency↓ Sleep disturbances↓ Daytime dysfunction↓	5
Cody et al. (51)	America	Two-arm RCT	Total:55.82 (4.34)	Total: 33	Unclear	(1) A newer version of the speed of processing program, including Double Decision and Target Tracker; (2) Received a 2.0 mA current for 20 min while engaging in the speed of processing training	PSQI	No significant changes in sleep outcomes	6

^*^Only the non-pharmacological intervention group based on sleep hygiene was included.

RCT, Randomized controlled trial; TRAZ, trazodone; SHT, sleep hygiene training; PLWH, people living with HIV; ISI, Insomnia Severity Index; PSQI, Pittsburgh Sleep Quality Index; PROMIS, patient-reported outcomes measurement system; SHPS, Sleep Hygiene Practice Scale; SDQ, sleep disturbance questionnaire; GSDS, General Sleep Disturbance Scale; ESS, Epworth Sleepiness Scale; CSQI, Current Sleep Quality Index; ↑, significantly improved; ↓, significantly decreased.

The mean age of the participants ranged from 35.7 to 61.03. The sample size varied from 22 to 272. The total number of participants was 1,132. Eight studies reported that all participants received ART (37, 39, 40, 42, 43, 45–47), and one study reported that 95% of participants received ART (38). Seven studies did not specify poor sleep as an inclusion criterion for participants (38, 39, 41–43, 49, 51), which provides evidence that non-pharmacological interventions do not disrupt normal sleep.

### 3.3 Description of the interventions

A summary of TIDieR checklists for all the included studies was provided in Supplementary material 2.

Following the classification of the Collaborative University Platform for Evaluating Health Prevention and Supportive Care Programs (52), the included non-pharmacological interventions were differentiated into three categories according to intervention contents, which were psychological (*n* = 6), physical (*n* = 8), and elemental health intervention (*n* = 1). The psychological interventions were mainly components of cognitive-behavioral therapy for insomnia (37–41) or mindfulness-based cognitive therapy (42). The physical intervention included an exercise program (43) and manual therapies based on acupuncture (44) or auricular plaster therapy (45–50). The elemental health intervention based on electromagnetics used the speed of processing (SOP) training with transcranial direct current stimulation (tDCS) to examine sleep changes in PLWH (51). Alikhani et al. (41) compared the impact of sleep hygiene and trazodone on the sleep of PLWH, and we only included and analyzed non-pharmacological interventions based on sleep hygiene. Hixon et al. (43) explored the effects of an exercise intervention on the sleep of older adults with and without HIV, and we only analyzed the sleep changes of PLWH groups.

One study reported intervention development based on socio-ecological theories (38). Although part of the studies reported the qualifications of intervention providers (37–41, 44, 46, 48), none of the studies described their expertise and any relevant training received. The interventions of three studies were delivered in a group or an individual (41, 42, 44). Eight studies described how the intervention was tailored individually (37, 39, 43–45, 48, 49, 51), and two studies reported the intervention modification (37, 44). Most studies have reported acceptable retention rates for interventions (37–41, 43, 44, 46–48, 50, 51), and three studies have described the strategies used to maintain or improve fidelity (37, 38, 40).

### 3.4 Sleep measurements

In the included studies, the sleep measurements can be divided into objective measures via actigraphy and subjective patient-reported outcomes including sleep diaries and scales (53). First, wrist actigraphy was used to measure sleep index objectively, such as sleep efficiency, total sleep time, sleep latency, and wake-up time after sleep (38, 39, 43, 44). Second, sleep diaries were used to record daily activities, bedtimes and wake times, wake after sleep onset, and self-rated sleep quality (37). Self-reported scales were used to measure sleep in PLWH, including Pittsburgh Sleep Quality Index (PSQI) or its subscales (37, 39–51), Insomnia Severity Index (ISI) (37, 41), Patient-Reported Outcomes Measurement System (PROMIS) Sleep Disturbance and Sleep-Related Impairment (37, 38), General Sleep Disturbance Scale (GSDS) (39), Epworth Sleepiness Scale (ESS) (41), Current Sleep Quality Index (CSQI) (44), Sleep Hygiene Practice Scale (SHPS) (37) and Sleep Disturbance Questionnaire (SDQ) (37), which could measure participants' sleep quality, sleep disturbances, and daytime sleepiness. Seven studies provided data on the reliability of these scales, whether calculated in previous studies or in the studies they conducted (38–42, 44, 51). Only two studies had a week (37) or 2- week (46) follow-up period, and all other studies measured sleep outcomes immediately after the intervention. No study reported how to deal with missing sleep data.

### 3.5 Impact

Details of the impact of these interventions on sleep-related outcomes are shown in Table 1.

#### 3.5.1 Psychological interventions

The psychological interventions were designed according to components of cognitive-behavioral therapy for insomnia (37–41) or mindfulness-based cognitive therapy (42). The findings showed statistically significant improvements in subjective and objective sleep outcomes except for one study (38). The perceived severity of insomnia symptoms measured by ISI was reduced (37), and sleep quality measured by PSQI improved (37, 40–42). Additionally, total sleep time, sleep efficacy, and self-rated sleep quality recorded in sleep diaries increased, while sleep onset latency and sleep-wake after sleep onset decreased (37). The objective results measured by wrist actigraphy revealed an increase in sleep maintenance for poor sleepers (39).

#### 3.5.2 Physical interventions

Six studies evaluated the effects of auricular plaster therapy (APT) on sleep in PLWH (45–50), and the acupressure techniques differed slightly among the studies. APT employed the pressing method of primary acupoints plus auxiliary acupoints. Results showed significant increases in overall and subscale PSQI scores after the intervention (37, 38, 45–50), indicating that APT could improve sleep quality, sleep efficiency, sleep duration and reduce sleep latency, sleep disturbances, medication use, and daytime dysfunction for PLWH. Phillips and Skelton conducted a pretest/posttest experimental design to examine the effects of individualized acupuncture on the sleep of PLWH (44), which showed that the subjective sleep quality of PLWH measured by CSQI was significantly improved from the pretest to the posttest. The objective sleep indices measured by wrist actigraphy showed that the total sleep time increased by an average of 2 h per day after intervention, and the amount of sleep in minutes, sleep percentage, and sleep ratio increased significantly, while the number of minutes spent awake and wake percentage decreased significantly.

Another physical intervention is an exercise program for sedentary older adults with and without HIV (43). According to the eligibility criteria, we only included the HIV-infected group in this review. Participants received a 24-week supervised exercise intervention with gradually increasing exercise intensity, but the subjective and objective sleep did not improve.

#### 3.5.3 Elemental intervention

Cody et al. randomly assigned older adults with HIV to receive twice-a-week speed of processing training with tDCS or speed of processing training with sham tDCS for 5 weeks (51). There was no significant difference in global PSQI scores between the sham tDCS group and the active tDCS group.

### 3.6 Risk of bias

Detailed risk of bias assessment is presented in Supplementary material 3, and assessment scores are also presented in Table 1. For the nine included RCTs, none of the interveners in the studies were blind to treatment assignment. Only two studies (38, 46) conducted blinding methods of treatment assignment for outcome assessors or study subjects. The reliability of sleep measures and the appropriateness of statistical analysis were at high risk in one study (45), and the methodological quality of other studies was at low risk.

## 4 Discussion

To our best knowledge, this is the first systematic narrative review identifying and summarizing the available empirical evidence of non-pharmacological interventions for improving sleep in PLWH and providing a detailed summary of the content of these interventions and the impact of improving sleep in PLWH. This review extracted 15 interventions, covering three function categories of psychological, physical, and elemental health intervention. By revealing intervention content and details, this review provides translational evidence to improve intervention design, research quality, and clinical service delivery.

Consistent with previous evidence (54), six psychological interventions modified based on CBT-I or mindfulness cognitive therapy improved the sleep of PLWH to varying degrees statistically and clinically. Our findings align with the recent systematic review of nurse-led symptom management in PLWH (27), which found that social-behavioral interventions were well accepted and favorably received by PLWH, but the feasibility and efficacy of these educational interventions to improve sleep and fatigue remain uncertain. Although our review showed the preliminary efficacy of psychological interventions for PLWH, caution should be exercised in interpreting the findings because most of the studies included in this review were feasibility or pilot studies with small sample sizes, limited power, and a lack of control groups. CBT-I consists of sleep hygiene/education, stimulus control, sleep restriction, relaxation therapy, and cognitive therapy, which can correct participants' wrong perception of sleep disturbance and inappropriate behavioral factors, eliminate psychophysiological hyperarousal, and enhance sleep drive (20, 55). In addition, the relaxation method in CBT-I and the meditation method in mindfulness could clear the bothersome mind and create a path to inner peace (23). The European Sleep Research Society claims that CBT-I has better long-term efficacy than hypnotics and recommends it as a first-line treatment for chronic insomnia in adults (24). The five included CBT-I studies all employed sleep behavioral strategies (37–41), and none of them used cognitive components. Although the exact efficacy of each component may differ, evidence shows that a package of care is more effective for sleep improvement than separate cognitive or behavioral components (56), suggesting that future research should tailor a package of intervention strategies according to the sleep problems of the participants.

Our work reviews the physical interventions for improving sleep in PLWH comprehensively. APT and acupuncture both belong to manual therapy in physical intervention. Manual therapy is a non-surgical type of conservative management that includes different skilled hands/fingers-on techniques directed to the patient's body to assess, diagnose, and treat various symptoms and conditions (57). All APTs included in this review showed improved self-reported sleep quality in PLWH. Similarly, the APT has been proven effective for insomnia in multiple clinical populations (58, 59). However, due to low-quality evidence, the European Sleep Research Society does not recommend APT as a treatment for insomnia (24). All included APT studies in this review had acceptable literature quality. The mechanism of APT to improve sleep includes regulating the nervous system and neurotransmitters (60). On the one hand, APT alleviates insomnia by increasing cardiac parasympathetic activity and decreasing sympathetic activity (61). On the other hand, APT improves sleep by increasing gamma-aminobutyric acid and nocturnal melatonin secretion (62, 63). The acupoints of most included APTs are individually selected based on the participants' symptoms and thus targeted to alleviate the participants' symptoms of sleep disturbances. Acupuncture is another commonly used physical intervention, and the included research showed that individualized acupuncture programs could significantly improve the quality and quantity of sleep in PLWH (44). HIV is a blood-borne virus. Therefore, the safety of acupuncture in treating patients with HIV/AIDS and insomnia should be a key consideration. How to correctly handle contaminated sharps such as needles and ensure the safety of the trial is a significant challenge for researchers and clinicians.

We described the non-pharmacological interventions in sufficient detail using the TIDieR checklist to compare the characteristics of these interventions, highlight missing or unavailable details, and identify the interventions that might be implemented in different settings (28). Unlike pharmacotherapies and surgeries, which have specific targets and unique action mechanisms, non-pharmacological interventions simultaneously mobilize a cascade of several biopsychosocial mechanisms (23). Program theory helps explain how and under what conditions specific interventions lead to the expected outcomes. However, only one study developed interventions based on socio-ecological theory (38), so understanding the mechanisms of how interventions cause changes remains poor. Non-pharmacological interventions can be tailored according to participants' situations and preferences (28). CBT-I adjusts bedtimes and rise times according to participants' daily schedules (37), and APT selects the primary and auxiliary acupoints according to the participants' symptoms (45, 48, 49). Tailored interventions meet individualized needs and are well accepted by participants. Hence, future studies should provide a brief rationale and guide for intervention tailoring in PLWH. Reports on the eligibility criteria of intervention providers and centers remain poor among included studies, which may influence the applicability of the trial results. The included RCTs were at high risk of whether the participants, intervention providers, and those outcomes assessors were blinded to group assignment, which may lead to the selection and information bias and affect the authenticity of results. Although blinding methods are complicated in non-pharmacological interventions, the blinding issues associated with the feasibility of blinding, the risk of blinding failure, and the risk of bias when blinding is not feasible should be discussed (28). Understanding the intervention details of the available evidence helps pave the way for future research and informs sleep disturbance treatment guidelines for PLWH. Given the poor reporting of the details of existing non-pharmacological interventions, more high-quality, well-described original intervention studies are needed in the future.

Subjective measures via scales and sleep diaries and objective measures via actigraphy have been used to assess the impact of non-pharmacological interventions on sleep in PLWH. Psychological and physical interventions can improve subjective sleep and the amount of objective sleep measured by actigraphy in PLWH, including increased total sleep time and decreased nighttime waking time. PSQI is the most commonly used scale for measuring subjective self-perceived sleep in PLWH, which measures seven aspects of sleep, including subjective sleep quality, sleep latency, sleep duration, sleep efficiency, sleep disturbances, sleep medication use, and daytime dysfunction (64). PSQI has been proven highly correlated with polysomnography (64) and has good reliability and validity in PLWH (65). Patient-reported outcomes measured by scales or questionnaires are often recommended to describe individual feelings and long-term complaints better than short-term objective measurements (66). However, some included studies did not report the reliability and validity of the measurement tools and how to deal with missing data statistically, which affected the interpretation and inference of the results (67). Sleep diaries were another widely used tool for collecting data over time on self-reported sleep and related function. However, the lack of a standardized sleep diary compromised the ability to fully interpret and integrate the results of previous findings (68). Wrist actigraphy, the most commonly used device for measuring objective sleep quantity and structure, allows participants to measure sleep in their familiar environment, is more convenient and less expensive than polysomnography, and sleep parameters measured by wrist actigraphy are highly associated with polysomnography (44, 69). However, actigraphy might be discontinued in PLWH due to the loss of the device during the intervention (37). Future research might improve the feasibility of sleep measurement devices by simplifying them. Although the results of subjective and objective sleep measures were consistent in this review, discrepancies between subjective and objective sleep indicators frequently occur in people with poor sleep (70, 71). Subjective and objective measurements of sleep help to characterize sleep disturbances, and we recommend the combination of objective and subjective measurements, including well-recognized reliable and valid scales, structured clinical sleep interviews, consensus sleep diaries, and wrist actigraphy, to comprehensively assess the sleep quality and quantity of PLWH, and accurately identify the characteristics of sleep disturbances in this group and the dynamic changes of sleep before and after the intervention. Since the sleep measurement tools used in the included studies were generic and did not reflect the specific characteristics of sleep in PLWH, developing specific sleep measurement tools for PLWH is conducive to gaining insight into sleep problems in this population.

### 4.1 Limitations and implications

Several limitations of this review should be noted. First, given the incomplete description of intervention details and the lack of meta-analysis, caution should be exercised in concluding the effectiveness of non-pharmacological interventions to improve sleep in PLWH. Second, most of the existing non-pharmacological interventions for AIDS patients focus on sleep behavior habits and insomnia symptoms but do not consider the specific factors that cause sleep disorders in AIDS patients, such as high viral load (13), low CD4^+^ count (14), depression (6), HIV-related stigma (8), etc. In the future, the development of sleep interventions should fully consider HIV/AIDS-related specific diseases and psychological and behavioral factors. Third, we only included published studies from peer-reviewed journals without exploring unpublished gray literature, which might affect the final findings and bias the data toward positive results. Finally, to make the conclusion more focused, we excluded studies not aimed at improving sleep, such as interventions targeting improving fatigue and cognitive function that have also been shown to be effective in improving sleep in PLWH (72, 73). Future studies can integrate such intervention strategies and test their effects.

Despite the limitations, our work gives a whole picture of the non-pharmacological sleep promotion interventions for HIV-infected people. This review shows that components of cognitive-behavioral therapy for insomnia and auricular plaster therapy have improved the sleep of PLWH, which provides evidence for the clinical management of sleep disturbances during AIDS care. Future non-pharmacological interventions follow protocols with evidence-based dosing, contents, and measures to ensure their sustainable and significant effects.

## 5 Conclusion

This review gives a whole picture of the non-pharmacological interventions for improving sleep in PLWH. Our work shows that cognitive behavioral therapy for insomnia as a psychological intervention and auricular plaster therapy as a physical intervention have the potential to improve sleep in PLWH, and the combination of patient-reported outcomes and simple devices might help comprehensively assess sleep. Due to the limited quality and quantity of studies, more high-quality RCTs are needed to test the effectiveness and implementation of non-pharmacological interventions.

## Data availability statement

The original contributions presented in the study are included in the article/Supplementary material, further inquiries can be directed to the corresponding author.

## Author contributions

JM and MW designed the review. JM and CZ conducted the systematic literature search, study selection, and data collection supervised by HW. JM and HW performed the critical evaluation of bias supervised by CZ. JM wrote the first draft of the manuscript with supervision from MV. MW and MV revised the first draft of the manuscript and commented on drafts. All authors contributed extensively to the work presented in this article, and approved the final version.
