# Supplementary material for: Non-pharmacological interventions for improving sleep in people living with HIV: a systematic narrative review
Source: Front Neurol. 2023 Nov 20;14:1017896. doi: 10.3389/fneur.2023.1017896 (PMC10732507; doi:10.3389/fneur.2023.1017896)
Supplement: Supplementary file 1 [file Table_1.DOCX]

**PubMed**

#1 HIV Infections[MeSH] OR HIV Infections[tw] OR HIV[MeSH] OR HIV[tw] OR hiv‐1*[tw] OR hiv‐2*[tw] OR hiv1[tw] OR hiv2[tw] OR HIV infect*[tw] OR human immunodeficiency virus[MeSH] OR human immunodeficiency virus[tw] OR human immunedeficiency virus[tw] OR human immuno‐deficiency virus[tw] OR human immune‐deficiency virus[tw] OR ((human immun*) AND (deficiency virus[tw])) OR acquired immunodeficiency syndrome[MeSH] OR AIDS OR acquired immunodeficiency syndrome[tw] OR acquired immunedeficiency syndrome[tw] OR acquired immuno‐deficiency syndrome[tw] OR acquired immune‐deficiency syndrome[tw] OR ((acquired immun*) AND (deficiency syndrome[tw])) OR HIV Seropositivity[MeSH] OR HIV Seropositivity[tw] OR AIDS Serodiagnosis[MeSH] OR AIDS Serodiagnosis[tw] OR "sexually transmitted diseases, viral"[MeSH] OR "sexually transmitted diseases, viral"[tw] OR HIV/AIDS[tw]

#2 Sleep[MeSH] OR Sleep*[tw] OR Insomnia*[tw] OR Sleep disorder*[tw] OR Sleep disturbance*[tw] OR Sleep difficult*[tw] OR sleepless*[tw] OR Sleep problem[tw] OR Sleep quality[tw] OR Sleep Initiation and Maintenance Disorders[MeSH] OR Sleep Initiation and Maintenance Disorders[tw] OR Disorders of Initiating and Maintaining Sleep[tw] OR Sleep Arousal Disorders[MeSH] OR Sleep Arousal Disorders[tw] OR Sleep Disorders, Intrinsic[MeSH] OR Sleep Disorders, Intrinsic[tw] OR Sleep Paralysis[MeSH] OR Sleep Paralysis[tw] OR Sleep Stages[MeSH] OR Sleep Stages[tw] OR Sleep Wake Disorders[MeSH] OR Sleep Wake Disorders[tw] OR Sleep Deprivation[MeSH] OR Sleep Deprivation[tw] OR Dyssomnias[MeSH] OR Dyssomnias[tw] OR Parasomnias[MeSH] OR Parasomnias[tw] OR hypersomnia[tw] OR Wakefulness[MeSH] OR Wakefulness[tw] OR Arousal[MeSH] OR Arousal[tw] OR sleep disorders, circadian rhythm[MeSH] OR sleep disorders, circadian rhythm[tw] OR disorders of excessive somnolence[MeSH] OR disorders of excessive somnolence[tw] OR hypersomnolence*[tw] OR Sleepiness[MeSH] OR Sleepiness[tw]

#3 Non-pharmacological therapies[tw] OR Non-pharmacological intervention[tw] OR Complementary Therapies[MeSH] OR Complementary Therap*[tw] OR Alternative Therap*[tw] OR Music Therapy[MeSH] OR Music Therapy[tw] OR Sleep promotion[tw] OR Massage[MeSH] OR Massage[tw] OR Muscle Relaxation[MeSH] OR Muscle Relaxation[tw] OR Cognitive Behavioral Therapy[MeSH] OR Cognitive Behavioral Therapy[tw] OR Cognitive Therapy[tw] OR Behavior Therapy[MeSH] OR Behavior Therapy[tw] OR Behavior intervention[tw] OR Sleep Hygiene[MeSH] OR Sleep Hygiene[tw] OR Stimulus Control[tw] OR Sleep restriction[tw] OR Relaxation[MeSH] OR Relaxation[tw] OR Relaxation Therapy[MeSH] OR Relaxation Therapy[tw] OR Behavior Modification[tw] OR Psychotherapy[MeSH] OR Psychotherapy[tw] OR Aromatherapy[MeSH] OR Aromatherapy[tw] OR Physical Therapy Modalities[MeSH] OR Physical Therapy Modalities[tw] OR Acupuncture[MeSH] OR Acupuncture[tw] OR Acupoints massages[tw]

#4 #1 AND #2 AND #3

**Web of Science**

#1 TS=(“HIV Infections” OR HIV OR “hiv‐1” OR “hiv‐2” OR “hiv1” OR “hiv2” OR “HIV infect*” OR “human immunodeficiency virus” OR “human immunedeficiency virus” OR “human immuno‐deficiency virus” OR “human immune‐deficiency virus” OR “((human immun*) AND (deficiency virus))” OR “acquired immunodeficiency syndrome” OR AIDS OR “acquired immunedeficiency syndrome” OR “acquired immuno‐deficiency syndrome” OR “acquired immune‐deficiency syndrome” OR “((acquired immun*) AND (deficiency syndrome))” OR “HIV Seropositivity” OR “AIDS Serodiagnosis” OR “sexually transmitted diseases, viral” OR HIV/AIDS)

#2 TS=(Sleep* OR Insomnia* OR “Sleep disorder*” OR “Sleep disturbance*” OR “Sleep difficult*” OR sleepless* OR “Sleep problem” OR “Sleep quality” OR “Sleep Initiation and Maintenance Disorders” OR “Disorders of Initiating and Maintaining Sleep” OR “Sleep Arousal Disorders” OR “Sleep Disorders, Intrinsic” OR “Sleep Paralysis” OR “Sleep Stages” OR “Sleep Wake Disorders” OR “Sleep Deprivation” OR “Sleep Apnea Syndromes” OR Dyssomnias OR Parasomnias OR hypersomnia OR Wakefulness OR Arousal OR “sleep disorders, circadian rhythm” OR “disorders of excessive somnolence” OR hypersomnolence* OR Sleepiness)

#3 TS=(“Non-pharmacological therapies” OR “Non-pharmacological intervention” OR “Complementary Therapies” OR “Alternative Therap*” OR “Music Therapy” OR “Sleep promotion” OR Massage OR “Muscle Relaxation” OR “Cognitive Behavioral Therapy” OR “Cognitive Therapy” OR “Behavior Therapy” OR “Behavior intervention” OR “Sleep Hygiene” OR “Stimulus Control” OR “Sleep restriction” OR Relaxation OR “Relaxation Therapy” OR “Behavior Modification” OR Psychotherapy OR Aromatherapy OR “Physical Therapy Modalities” OR Acupuncture OR “Acupoints massages”)

#4 #1 AND #2 AND #3

**Embase**

#1 'human immunodeficiency virus infection'/exp OR 'human immunodeficiency virus'/exp OR 'acquired immune deficiency syndrome'/exp

#2 ‘HIV Infections’ OR HIV OR ‘hiv‐1’ OR ‘hiv‐2’ OR ‘hiv1’ OR ‘hiv2’ OR ‘HIV infect*’ OR ‘human immunodeficiency virus’ OR ‘human immunedeficiency virus’ OR ‘human immuno‐deficiency virus’ OR ‘human immune‐deficiency virus’ OR ‘((human immun*) AND (deficiency virus))’ OR ‘acquired immunodeficiency syndrome’ OR AIDS OR ‘acquired immunedeficiency syndrome’ OR ‘acquired immuno‐deficiency syndrome’ OR ‘acquired immune‐deficiency syndrome’ OR ‘((acquired immun*) AND (deficiency syndrome))’ OR ‘HIV Seropositivity’ OR ‘AIDS Serodiagnosis’ OR ‘sexually transmitted diseases, viral’

#3 'sleep'/exp OR insomnia/exp OR ‘Sleep disorder’/exp OR ‘sleep quality’/exp OR ‘sleep arousal disorder’/exp OR ‘sleep stage’/exp OR ‘sleep disordered breathing’/exp OR ‘sleep deprivation’/exp OR ‘parasomnia’/exp OR ‘hypersomnia’/exp OR ‘wakefulness’/exp OR ‘arousal’/exp OR ‘circadian rhythm sleep disorder’/exp OR ‘somnolence’/exp

#4 Sleep* OR Insomnia* OR ‘Sleep disorder*’ OR ‘Sleep disturbance*’ OR ‘Sleep difficult*’ OR sleepless* OR ‘Sleep problem’ OR ‘Sleep quality’ OR ‘Sleep Initiation and Maintenance Disorders’ OR ‘Disorders of Initiating and Maintaining Sleep’ OR ‘Sleep Arousal Disorders’ OR ‘Sleep Disorders, Intrinsic’ OR ‘Sleep Paralysis’ OR ‘Sleep Stages’ OR ‘Sleep Wake Disorders’ OR ‘Sleep Deprivation’ OR ‘Sleep Apnea Syndromes’ OR Dyssomnias OR Parasomnias OR hypersomnia OR Wakefulness OR Arousal OR ‘sleep disorders, circadian rhythm’ OR ‘disorders of excessive somnolence’ OR hypersomnolence* OR Sleepiness

#5 ‘alternative medicine’/exp OR ‘music therapy’/exp OR ‘Muscle Relaxation’/exp OR ‘cognitive behavioral therapy’/exp OR ‘cognitive therapy’/exp OR ‘Behavior Therapy’/exp OR ‘Sleep Hygiene’/exp OR ‘relaxation training’/exp OR ‘behavior modification’/exp OR ‘Psychotherapy’/exp OR ‘Aromatherapy’/exp OR ‘physiotherapy’/exp OR ‘acupuncture’/exp OR ‘acupoint’/exp

#6 ‘Non-pharmacological therapies’ OR ‘Non-pharmacological Intervention’ OR ‘Complementary Therapies’ OR ‘Alternative Therap*’ OR ‘Music Therapy’ OR ‘Sleep promotion’ OR Massage OR ‘Muscle Relaxation’ OR ‘Cognitive Behavioral Therapy’ OR ‘Cognitive Therapy’ OR ‘Behavior Therapy’ OR ‘Behavior Intervention’ OR ‘Sleep Hygiene’ OR ‘Stimulus Control’ OR ‘Sleep restriction’ OR Relaxation OR ‘Relaxation Therapy’ OR ‘Behavior Modification’ OR Psychotherapy OR Aromatherapy OR ‘Physical Therapy Modalities’ OR Acupuncture OR ‘Acupoints massages’

#7 #1 OR #2

#8 #3 OR #4

#9 #5 OR #6

#10 #7 AND #8 AND #9

**Cochrane Central Registry of Controlled Trials (CENTRAL)**

#1 [mh “HIV Infections”] OR [mh HIV] OR [mh “human immunodeficiency virus”] OR [mh “acquired immunodeficiency syndrome”] OR [mh “HIV Seropositivity”] OR [mh “sexually transmitted diseases, viral”] OR “HIV Infections” OR HIV OR “hiv‐1*” OR “hiv‐2*” OR hiv1 OR hiv2 OR “HIV infect*” OR “human immunodeficiency virus” OR “human immunedeficiency virus” OR “human immuno‐deficiency virus” OR “human immune‐deficiency virus” OR “((human immun*) AND (deficiency virus))” OR AIDS OR “acquired immunodeficiency syndrome” OR “acquired immunedeficiency syndrome” OR “acquired immuno‐deficiency syndrome” OR “acquired immune‐deficiency syndrome” OR “((acquired immun*) AND (deficiency syndrome))” OR “HIV Seropositivity” OR “AIDS Serodiagnosis” OR “sexually transmitted diseases, viral”

#2 [mh Sleep] OR [mh “Sleep Arousal Disorders”] OR [mh “Sleep Paralysis”] OR [mh “Sleep Disorders, Intrinsic”] OR [mh “Sleep Stages”] OR [mh “Sleep Wake Disorders”] OR [mh “Sleep Deprivation”] OR [mh Dyssomnias] OR [mh Parasomnias] OR [mh Wakefulness] OR [mh Arousal] OR [mh “disorders of excessive somnolence”] OR [mh Sleepiness] OR Sleep* OR Insomnia* OR “Sleep disorder*” OR “Sleep disturbance*” OR “Sleep difficult*” OR sleepless* OR “Sleep problem” OR “Sleep quality” OR “Sleep Initiation and Maintenance Disorders” OR “Sleep Initiation and Maintenance Disorders” OR “Disorders of Initiating and Maintaining Sleep” OR “Sleep Arousal Disorders” OR “Sleep Disorders, Intrinsic” OR “Sleep Paralysis” OR “Sleep Stages” OR “Sleep Wake Disorders” OR “Sleep Deprivation” OR Dyssomnias OR Parasomnias OR hypersomnia OR Wakefulness OR Arousal OR “sleep disorders, circadian rhythm” OR “disorders of excessive somnolence” OR hypersomnolence* OR Sleepiness

#3 [mh “Complementary Therapies”] OR [mh “Music Therapy”] OR [mh Massage] OR [mh “Cognitive Behavioral Therapy”] OR [mh “Muscle Relaxation”] OR [mh “Behavior Therapy”] OR [mh “Sleep Hygiene”] OR [mh Relaxation] OR [mh “Relaxation Therapy”] OR [mh Psychotherapy] OR [mh Aromatherapy] OR [mh “Physical Therapy Modalities”] OR [mh Acupuncture] OR “Nonpharmacological therapies” OR “Nonpharmacological intervention” OR “Complementary Therap*” OR “Alternative Therap*” OR “Music Therapy” OR “Sleep promotion” OR Massage OR “Muscle Relaxation” OR “Cognitive Behavioral Therapy” OR “Cognitive Therapy” OR “Behavior Therapy” OR “Behavior intervention” OR “Sleep Hygiene” OR “Stimulus Control” OR “Sleep restriction” OR Relaxation OR “Relaxation Therapy” OR “Behavior Modification” OR Psychotherapy OR Aromatherapy OR “Physical Therapy Modalities” OR Acupuncture OR “Acupoints massages”

#4 #1 AND #2 AND #3

**Cumulative Index to Nursing and Allied Health Literature (CINAHL)**

#1 (MH "Acquired Immunodeficiency Syndrome") OR (MH "HIV Infections") OR (MH "HIV Seropositivity") OR (MH "Human Immunodeficiency Virus") OR (MH "AIDS Serodiagnosis") OR Acquired Immunodeficiency Syndrome OR HIV Infections OR HIV Seropositivity OR Human Immunodeficiency Virus OR AIDS Serodiagnosis OR HIV OR AIDS OR hiv‐1* OR hiv‐2* OR hiv1 OR hiv2 OR HIV infect* OR human immuno‐deficiency virus OR human immune‐deficiency virus OR AIDS OR acquired immuno‐deficiency syndrome OR acquired immune‐deficiency syndrome OR HIV/AIDS

#2 (MH "Sleep") OR (MH "Parasomnias") OR (MH "Sleep Disorders") OR (MH "Dyssomnias") OR (MH "Sleep Stages") OR (MH "Sleep Deprivation") OR (MH "Insomnia") OR (MH "Wakefulness") OR (MH "Sleepiness") OR (MH "Sleep Arousal Disorders") OR (MH "Sleep Disorders, Intrinsic") OR (MH "Sleep Stages") OR (MH "Sleep Disorders, Circadian Rhythm") OR "Dyssomnias" OR (MH "Parasomnias") OR (MH "Wakefulness") OR (MH "Arousal") OR (MH "Disorders of Excessive Somnolence") OR (MH "Sleep Disorders, Circadian Rhythm") OR Sleep OR Parasomnias OR Sleep Disorders OR Dyssomnias OR Sleep Stages OR Sleep Deprivation OR Insomnia OR Wakefulness OR Sleepiness OR Sleep Arousal Disorders OR Insomnia* OR Sleep disorder* OR Sleep disturbance* OR Sleep difficult* OR Sleep problem OR Sleep quality OR Sleep Initiation and Maintenance Disorders OR Sleep Arousal Disorders OR Sleep Disorders, Intrinsic OR Sleep Stages OR Sleep Disorders, Circadian Rhythm OR Sleep Deprivation OR Dyssomnias OR Parasomnias OR Wakefulness OR Arousal OR Disorders of Excessive Somnolence OR Sleep Wake Disorders OR hypersomnia OR Sleep Disorders, Circadian Rhythm

#3 (MH "Alternative Therapies") OR (MH "Music Therapy") OR (MH "sleep promotion") (MH "Massage+") OR (MH "Muscle Relaxation") OR (MH "Behavior Therapy+") OR (MH "Cognitive Therapy+") OR (MH "Sleep Hygiene") OR (MH "Relaxation") OR (MH "Psychotherapy+") OR (MH "Aromatherapy") OR (MH "Physical Therapy") OR (MH "Behavior Modification") OR (MH "Psychotherapy") OR (MH "Aromatherapy") OR (MH "Acupuncture") OR Alternative Therapies OR Music Therapy OR Sleep promotion OR Massage OR Muscle Relaxation OR Behavior Therapy OR Cognitive Therapy OR Sleep Hygiene OR Relaxation OR Psychotherapy OR Aromatherapy OR Physical Therapy OR Behavior Modification OR Acupuncture OR Non-pharmacological therapies OR Non-pharmacological intervention OR Complementary Therap* OR Cognitive Behavioral Therapy OR Behavior intervention OR Stimulus Control OR Sleep restriction OR Psychotherapy OR Aromatherapy OR Physical Therapy Modalities OR Acupuncture OR Acupoints massages

#4 #1 AND #2 AND #3

**Clinical trails**

#1 HIV OR human immunodeficiency virus OR AIDS OR acquired immunodeficiency syndrome

#2 Sleep OR Insomnia OR Sleep disorder OR Sleep disturbance OR sleepless OR Sleep problem OR Sleep quality OR Sleep Initiation and Maintenance Disorders OR Disorders of Initiating and Maintaining Sleep OR Sleep Arousal Disorders OR Sleep Disorders, Intrinsic OR Sleep Paralysis OR Sleep Stages OR Sleep Wake Disorders OR Sleep Deprivation OR Dyssomnias OR Parasomnias OR hypersomnia OR Wakefulness OR Arousal OR sleep disorders, circadian rhythm OR disorders of excessive somnolence OR hypersomnolence OR Sleepiness

#3 Non-pharmacological therapies OR Non-pharmacological intervention OR Complementary Therapies OR Alternative Therapies OR Music Therapy OR Sleep promotion OR Massage OR Muscle Relaxation OR Cognitive Behavioral Therapy OR Cognitive Therapy OR Behavior Therapy OR Behavior intervention OR Sleep Hygiene OR Stimulus Control OR Sleep restriction OR Relaxation OR Relaxation Therapy OR Behavior Modification OR Psychotherapy OR Aromatherapy OR Physical Therapy Modalities OR Acupuncture OR Acupoints massages

#4 #1 AND #2 AND #3

**China National Knowledge Infrastructure (CNKI)**

# 1 艾滋病 OR 获得性免疫缺陷综合征 OR HIV OR AIDS

# 2 睡眠 OR 睡眠障碍 OR 失眠

# 3 # 1 AND # 2

**Wanfang Data**

#1 艾滋病 OR 获得性免疫缺陷综合征 OR HIV OR AIDS

#2 睡眠 OR 睡眠障碍 OR 失眠

# 3 # 1 AND # 2

**China Biology Medicine disc (CBM)**

#1 (“获得性免疫缺陷综合征”[不加权: 扩展]) OR “HIV” [不加权: 扩展]

#2 (((“睡眠”[不加权: 扩展]) OR “入睡和睡眠障碍”[不加权: 扩展]) OR “失眠症”[不加权: 扩展]) OR “睡眠异常”[不加权: 扩展]

#3 艾滋病 OR 获得性免疫缺陷综合征 OR HIV OR AIDS

#4 睡眠 OR 入睡和睡眠障碍 OR 失眠症 OR 睡眠异常 OR 失眠 OR 睡眠障碍

#5 #3 OR #1

#6 #4 OR #2

#7 #6 AND #5
