# Supplementary material for: Non-pharmacological interventions for improving sleep in people living with HIV: a systematic narrative review
Source: Front Neurol. 2023 Nov 20;14:1017896. doi: 10.3389/fneur.2023.1017896 (PMC10732507; doi:10.3389/fneur.2023.1017896)
Supplement: Supplementary file 2 [file Table_2.docx]

Table 2 Description of the interventions (modified based on TIDieR checklists and guide)

| Author, year | Category | Brief name | Why  (Theory or goals) | What  (Materials) | What  (Procedures) | Who provided | How | Where | When and how much | Tailoring | Modifications | How well (Planned strategies to maintain/improve fidelity or actual fidelity) |
| --- | --- | --- | --- | --- | --- | --- | --- | --- | --- | --- | --- | --- |
| Buchanan et al., 2018 | Psychological | BBTI (Brief Behavioral Treatment for Insomnia) | Goal: To test the feasibility, acceptability, and initial efficacy of BBTI, particularly to explore population-specific patterns contributing to sleep difficulties | A BBTI workbook | Focus on the behavioral components of insomnia treatment, particularly sleep education and discussion of homeostatic and circadian mechanisms of sleep regulation, including (a) reduced time in bed; (b) keeping the same rise time regardless of sleep quality; (c) going to bed only when sleepy; (d) getting out of bed during the night when not asleep; (e) sleep hygiene | Clinical nurses | Two in-person (Sessions 1 and 3) and two by telephone (Sessions 2 and 4); | Clinic room | Four weekly sessions | Time in bed was adjusted according to the participants’ total sleep time, sleep onset latency, and wake after sleep onset | Abandoning the use of wrist actigraphy due to the loss of equipment | Planned: wait-list design; randomization after completion of Week 1 pretreatment assessments.  Actual retention rate: 54.5% (12/22) |
| Webel et al., 2013 | Psychological | SystemCHANGE–HIV intervention | Theory: Socioecological theory;  Goal: To evaluate the feasibility and estimate the magnitude of the effect of a novel, evidence-based behavioral intervention on improving sleep outcomes in PLWH | A semi-scripted lesson plan for facilitators | Redesign the daily system of the interpersonal environment, routines, and events linked to sleep behaviors, including sleep hygiene, and behavioral modification strategies | A trained health educator or a registered nurse | Face to face | A convenient community-based setting | Ten weekly sessions | Not reported | Not reported | Planned: (a) The facilitators used a semi-scripted lesson plan to increase the fidelity of information delivered; (b) The intervention group was reminded to come to each session by a weekly phone call from the study staff; (c) Using a participant feedback form to assess the intervention fidelity at the end of each session.  Actual retention rate: 95.2% (20/21);  On average, participants attended 71% of all intervention sessions |
| Hudson et al., 2008 | Psychological | A tailored sleep promotion intervention | Goal: To test the efficacy of a tailored sleep promotion intervention protocol based on principles of sleep hygiene | Participants received a 30- to 45-min session in which six primary principles of sleep hygiene were reviewed | The intervention was an educational and behavioral set of sleep-promoting behaviors based on principles of sleep hygiene, including the rules of a bedroom, exercise, tension, time to sleep, eating/drinking/drugs, and rhythm | Healthcare provider | Face to face | Clinic settings | One time a 30- to 45-min session | Participants were required to try two of the potential six sleep hygiene behaviors based on the review of individual sleep behaviors | Not reported | Actual retention rate: 93.75% (30/32) |
| Dreher, 2003 | Psychological | Caffeine reduction | Goal: To verify whether reducing caffeine intake by 90% can improve the sleep quality of PLWH | Caffeine tracking diary, which incorporated both a 24-h diary and weekly estimation of caffeine with precise measurements | Participants were instructed first to withdraw from caffeine using a Gradual Withdrawal from Caffeine Protocol and then asked to avoid all caffeine sources for 30 days | A certified nutrition support nurse with expertise in HIV nutrition | Not reported | Participants were recruited from print sources and the Internet | 30 days caffeine reduction | Not reported | Not reported | Planned: all subjects were tracked twice (every two weeks) using a self-report Compliance Form to determine how adherent they believed they were to the requirement to abstain completely from caffeine or maintain their usual daily caffeine intake.  Actual retention rate: 66.7% (44/66) |
| Alikhani et al., 2020 | Psychological | Sleep hygiene training (SHT) | Goal: To improve the sleep of HIV patients undergoing methadone maintenance therapy | A sleep hygiene training | Sleep hygiene principles, such as “have a regular sleep schedule,” “get rid of ‘bad’ thoughts to fall asleep,” and “Dos and Don’ts before going to sleep” | A clinical psychologist experienced in sleep hygiene education | Face to face; 8 to 10 participants in each group | Clinic room | The training was conducted once a week, lasting 50-60 minutes for 12 weeks | Not reported | Not reported | Actual retention rate: 44% (11/25) |
| Molavi et al., 2020 | Psychological | Mindfulness-based cognitive therapy | Goal: To investigate the effectiveness of mindfulness-based cognitive therapy in the improvement of sleep quality | Mindfulness-based cognitive therapy training sessions | Mindfulness-based cognitive therapy training, including meditation, breathing exercises, body scanning, relaxation, Yoga exercises, metaphor therapy, and light communication exercises | Not reported | In a group | The behavioral disease counseling center | Eight weekly sessions, and each last 1.5 hours | Not reported | Not reported | Not reported |
| Hixon et al., 2020 | Physical | Supervised exercise intervention | Goal: To investigate the effect of an exercise intervention on the subjective and objective sleep of PLWH | Treadmill; Exercise machine | Moderate-intensity endurance and resistance exercises in the first 12 weeks and randomly performed moderate-intensity or high-intensity exercises in the next 12 weeks | Not reported | Not reported | Exercise research laboratory | Three times a week for 24 weeks | Exercise intensity was adjusted for baseline VO2 and 1-RM, and the choice of exercise intensity between 12 and 24 weeks was balanced by HIV serostatus, gender, and age | Not reported | Actual retention rate: 87.5% (28/32) |
| Phillips and Skelton, 2001 | Physical | Individualized Acupuncture | Goal: To test the effectiveness of acupuncture delivered in a group setting for improving sleep quality in PLWH | Sterile, disposable acupuncture needles | Patients sat in comfortable chairs in an ambient environment, with access to pillows for use behind their head or back, on their lap for their arms, or under their feet. Soft background music was played to facilitate relaxation. Needle insertion sites were prepared with alcohol before each treatment. Individualized acupuncture treatments that focused on specific needs and symptoms that the individual was experiencing | Certified acupuncturists | Face to face, in a group setting | AIDS support organization | Twice a week for five weeks for a total of 10 sessions | Each patient received individualized acupuncture treatments that focused on specific needs and symptoms that the individual was experiencing | The treatment was modified throughout the study to accommodate the individual’s changing sleep pattern, pain, or other health issues | Actual retention rate: 91.3% (21/23) |
| Chen et al., 2017 | Physical | Auricular plaster therapy | Goal: To observe the clinical effects of auricular plaster therapy for insomnia in PLWH | Bean | Pressing primary acupoints plus auxiliary acupoints | Not reported | Not reported | Not reported | One-sided acupoint selection 1-2 min/time, 4-5 times a day, alternate acupoints in both ears, exchange once every three days. 7 days is a course of treatment, and four courses of treatment are observed continuously, with an interval of 1 day between each course | The choice of auxiliary acupoints depends on the participant’s symptoms | Not reported | Not reported |
| Chen, 2018 | Physical | Auricular plaster therapy | Goal: To observe the clinical effects of auricular plaster therapy for insomnia in PLWH | Bean | Pressing primary acupoints plus auxiliary acupoints | A clinically trained nurse | Face to face; The nurse helped the participants put the beans and plaster on their auricular points, and the participants pressed the acupoints by themselves | Not reported | One-sided acupoint selection 2 min/time, 3-5 times a day, alternate acupoints in both ears, exchange once every three days. 7 days is a course of treatment, and four courses of treatment are observed continuously, with an interval of 1 day between each course | Not reported | Not reported | Actual: retention rate: 92.5% (37/40) |
| Y. Zhang, 2018 (a) | Physical | Auricular plaster therapy with emotional intervention | Goal: To explore the effects of e auricular plaster therapy combined with emotional intervention on sleep quality and negative emotion in patients with AIDS-related insomnia | Bean, music, movies | (a) Pressing primary acupoints plus auxiliary acupoints; (b) Instruct participants to vent their bad feelings (e.g., listening to cheerful music, reading positive materials, doing sports, watching movies, walking) | Not reported | Not reported | AIDS inpatient ward | (a) One-sided acupoint selection 1-2 min/time, 4-5 times a day, alternate acupoints in both ears, exchange once every three days. 7 days is a course of treatment, and four courses of treatment are observed continuously, with an interval of 1 day between each course. (b) emotional intervention: once a day for ten minutes | Not reported | Not reported | Actual retention rate: 100% (43/43) |
| Y. Zhang, 2018 (b) | Physical | Auricular plaster therapy | Goal: To explore the effects of e auricular plaster therapy on sleep quality and negative emotions in patients with AIDS-related insomnia | Bean | Pressing primary acupoints plus auxiliary acupoints | Not reported | Not reported | AIDS inpatient ward | One-sided acupoint selection 1-2 min/time, 4-5 times a day, alternate acupoints in both ears, exchange once every three days. 7 days is a course of treatment, and four courses of treatment are observed continuously, with an interval of 1 day between each course | Not reported | Not reported | Actual: retention rate: 100% (43/43) |
| Sun, 2019 (a) | Physical | Auricular plaster therapy with emotional intervention | Goal: To observe the clinical effects of auricular plaster therapy with emotional intervention for insomnia in PLWH | Bean, music | (a) Pressing primary acupoints; (b) The nurse chose the corresponding emotional intervention methods according to the patient's psychological symptoms, such as chatting, listening to music, disease education | (a) Auricular plaster therapy: not reported; (b) Emotional intervention: nurses | Not reported | Not reported | (a) One-sided acupoint selection 1-2 min/time, three times a day for 15 days, alternate acupoints in both ears, exchange once every three days; (b) emotional intervention: not reported | The nurse chose the corresponding emotional intervention methods according to the patient’s psychological symptoms | Not reported | Actual: retention rate: 100% (29/29) |
| Sun, 2019 (b) | Physical | Auricular plaster therapy | Goal: To observe the clinical effects of auricular plaster therapy for insomnia in PLWH | Bean | Pressing primary acupoints | Not reported | Not reported | Not reported | One-sided acupoint selection 1-2 min/time, three times a day for 15 days, alternate acupoints in both ears, exchange once every three days | Not reported | Not reported | Actual: retention rate: 100% (29/29) |
| L. Zhang, 2018 (a) | Physical | Auricular plaster therapy with emotional intervention | Goal:  To observe the clinical effects of auricular plaster therapy with emotional intervention for insomnia in PLWH | Bean, music | (a) Pressing primary acupoints plus auxiliary acupoints; (b) Using ways such as talking to patients and listening to music to help them vent their emotions and encourage them | Not reported | Not reported | Not reported | (a) One-sided acupoint selection 30 s-1 min/time, 3-5 times a day, alternate acupoints in both ears, exchange once every three days. 7 days is a course of treatment, and four courses of treatment are observed continuously, with an interval of 1 day between each course. (b) emotional intervention: not reported | The choice of auxiliary points depends on the participant’s symptoms | Not reported | Not reported |
| L. Zhang, 2018 (b) | Physical | Auricular plaster therapy | Goal: To observe the clinical effects of auricular plaster therapy for insomnia in PLWH | Bean | Pressing primary acupoints plus auxiliary acupoints | Not reported | Not reported | Not reported | One-sided acupoint selection 30s-1 min/time, 3-5 times a day, alternate acupoints in both ears, exchange once every three days. 7 days is a course of treatment, and four courses of treatment are observed continuously, with an interval of 1 day between each course. | The choice of auxiliary points depends on the participant’s symptoms | Not reported | Not reported |
| Li, 2021 | Physical | Auricular plaster therapy | Goal: To observe the clinical effect of auricular plaster therapy on elderly AIDS patients with insomnia | Bean | Pressing primary acupoints plus auxiliary acupoints | Not reported | The participants pressed the acupoints by themselves | Not reported | One-sided acupoint selection 2 min/time, three times a day, alternate acupoints in both ears, exchange once every three days. 7 days is a course of treatment, and four courses of treatment are observed continuously, with an interval of 1 day between each course | Not reported | Not reported | Actual: retention rate: 100% (68/68) |
| Cody et al, 2019 (a) | Elemental | Transcranial direct current stimulation (tDCS) with speed of processing (SOP) training | Goal: Examine changes in global sleep quality among older adults with HIV who received tDCS with SOP training | Games: Double Decision and Target Tracker; direct current stimulation machine | (a) 2.0 mA current for 20 minutes; (b) Double Decision: participants were presented with an object in the center of the computer monitor and a Route 66 sign in one of several peripheral fields at various speeds and then asked to identify which central object (car/truck) was presented and the location of the Route 66 sign; (c) Target Tracker: participants were presented one or more objects and asked to watch the objects as they moved across the screen among several other balls or jellyfish | Not reported | Face to face | Not reported | tDCS: 10 hours; SOP training: twice a week for 5 weeks | The tDCS amperage could be adjusted according to the participant’s body sensation | Not reported | Actual: retention rate: 78.6% (33/42) |
| Cody et al., 2019 (b) | Elemental | Sham tDCS with SOP training | Goal: Examine changes in global sleep quality among older adults with HIV who received sham tDCS with SOP training | Games: Double Decision and Target Tracker; direct current stimulation machine | (a) 2.0 mA current for only 30 seconds, and then the strength of the current ramped down to 0 mA; (b) Double Decision: participants were presented with an object in the center of the computer monitor and a Route 66 sign in one of several peripheral fields at various speeds, and then asked to identify which central object (car/truck) was presented and the location of the Route 66 sign; (c) Target Tracker: participants were presented one or more objects and asked to watch the objects as they moved across the screen among several other balls or jellyfish | Not reported | Face to face | Not reported | tDCS: 10 hours;  SOP training: twice a week for 5 weeks | Not reported | Not reported | Actual: total retention rate: 78.6% (33/42) |

TIDieR, template for intervention description and replication; PLWH, People living with HIV; tDCS, Transcranial direct current stimulation; SOP, speed of processing
