# Supplementary material for: Non-pharmacological interventions for improving sleep in people living with HIV: a systematic narrative review
Source: Front Neurol. 2023 Nov 20;14:1017896. doi: 10.3389/fneur.2023.1017896 (PMC10732507; doi:10.3389/fneur.2023.1017896)
Supplement: Supplementary file 3 [file Table_3.DOCX]

**JBI, 2017 critical appraisal checklist for Quasi-Experimental Study**

| Study, year | Item 1 | Item 2 | Item 3 | Item 4 | Item 5 | Item 6 | Item 7 | Item 8 | Item 9 | Score |
| --- | --- | --- | --- | --- | --- | --- | --- | --- | --- | --- |
| Buchanan et al., 2018 | Yes | Yes | NA | No | Yes | Yes | Yes | Yes | Yes | 7 |
| Hudson et al., 2008 | Yes | Yes | NA | No | Yes | Yes | Yes | Yes | Yes | 7 |
| Phillips and Skelton, 2001 | Yes | Yes | NA | No | Yes | Yes | Yes | Yes | Yes | 7 |
| Chen et al., 2017 | Yes | Yes | NA | No | No | Yes | Yes | No | No | 4 |
| Li, 2021 | Yes | Yes | NA | No | No | Yes | Yes | No | Yes | 5 |
| Hixon et al., 2020 | Yes | Yes | NA | No | Yes | Yes | Yes | Yes | Yes | 7 |

**JBI, 2017 critical appraisal checklist for RCT**

| Study, year | Item 1 | Item 2 | Item 3 | Item 4 | Item 5 | Item 6 | Item 7 | Item 8 | Item 9 | Item 10 | Item 11 | Item 12 | Item 13 | Score |
| --- | --- | --- | --- | --- | --- | --- | --- | --- | --- | --- | --- | --- | --- | --- |
| Webel et al., 2013 | Yes | Yes | Yes | Unclear | No | Yes | No | No | Yes | Yes | Yes | Yes | Yes | 9 |
| Dreher, 2003 | Yes | Unclear | No | No | NA | No | Yes | Yes | No | No | Yes | Yes | No | 5 |
| Alikhani et al., 2020 | Yes | No | Yes | No | No | No | Yes | Yes | Yes | Yes | Yes | Yes | Yes | 9 |
| Molavi et al., 2020 | Unclear | No | Yes | No | No | Unclear | Yes | Unclear | Yes | Yes | Yes | Yes | Yes | 7 |
| Chen, 2018 | Yes | Yes | Yes | Yes | No | Unclear | Yes | Yes | No | Yes | Yes | Yes | Yes | 10 |
| Y. Zhang, 2018 | No | No | Yes | No | No | No | Yes | Yes | Yes | Yes | Yes | Yes | Yes | 8 |
| Sun, 2019 | No | No | Yes | No | No | No | Yes | Yes | Yes | Yes | Yes | Yes | Yes | 8 |
| L. Zhang, 2018 | Yes | Unclear | Yes | No | No | Unclear | Yes | No | No | Yes | Yes | Yes | Yes | 7 |
| Cody, 2019 | Unclear | No | No | NA | NA | Unclear | Yes | No | Yes | Yes | Yes | Yes | Yes | 6 |
